# Phenotypic diversity of type III secretion system activity in enteropathogenic Escherichia coli clinical isolates

**DOI:** 10.1099/jmm.0.001907

**Published:** 2024-10-21

**Authors:** Carmen A. Contreras, Tracy H. Hazen, Carmen Guadarrama, Ramón Cervantes-Rivera, Theresa J. Ochoa, Pablo Vinuesa, David A. Rasko, Jose L. Puente

**Affiliations:** 1 Departamento de Microbiología Molecular, Instituto de Biotecnología, Universidad Nacional Autónoma de México, Cuernavaca, Mor., Mexico; 2 Programa de Medicina Humana, Universidad Privada Antenor Orrego, Trujillo, Peru; 3 Department of Microbiology and Immunology, Institute for Genome Sciences, University of Maryland School of Medicine, Baltimore, MD, USA; 4 Instituto de Medicina Tropical Alexander von Humboldt, Universidad Peruana Cayetano Heredia, Lima, Peru; 5 University of Texas School of Public Health, Houston, USA; 6 Programa de Ingeniería Genómica, Centro de Ciencias Genómicas, Universidad Nacional Autónoma de México, Cuernavaca, Mor., Mexico

**Keywords:** clinical isolates, enteropathogenic *Escherichia coli*, GrlA, Ler, locus of enterocyte effacement, pathogenesis, PerC, phenotypic diversity, regulatory mechanisms, type III secretion system

## Abstract

**Introduction.** Enteropathogenic *Escherichia coli* (EPEC) strains pose a significant threat as a leading cause of severe childhood diarrhoea in developing nations. EPEC pathogenicity relies on the type III secretion system (T3SS) encoded by the locus of enterocyte effacement (LEE), facilitating the secretion and translocation of bacterial effector proteins.

**Gap Statement.** While the regulatory roles of PerC (plasmid-encoded regulator) and GrlA (global regulator of LEE-activator) in *ler* expression and LEE gene activation are well-documented in the EPEC prototype strain E2348/69, understanding the variability in LEE gene expression control mechanisms among clinical EPEC isolates remains an area requiring further investigation.

**Aim.** This study aims to explore the diversity in LEE gene expression control mechanisms among clinical EPEC isolates through a comparative analysis of secretion profiles under defined growth conditions favouring either PerC- or GrlA-mediated activation of LEE expression.

**Methodology.** We compared T3SS-dependent secretion patterns and promoter expression in both typical EPEC (tEPEC) and atypical EPEC (aEPEC) clinical isolates under growth conditions favouring either PerC- or GrlA-mediated activation of LEE expression. Additionally, we conducted promoter reporter activity assays, quantitative real-time PCR and Western blot experiments to assess gene expression activity.

**Results.** Significant differences in T3SS-dependent secretion were observed among tEPEC and aEPEC strains, independent of LEE sequence variations or T3SS gene functionality. Notably, a clinical tEPEC isolate exhibited increased secretion levels under repressive growth conditions and in the absence of both PerC and GrlA, implicating an alternative mechanism in the activation of Ler (LEE-encoded regulator) expression.

**Conclusion.** Our findings indicate that uncharacterized LEE regulatory mechanisms contribute to phenotypic diversity among clinical EPEC isolates, though their impact on clinical outcomes remains unknown. This challenges the conventional understanding based on reference strains and highlights the need to investigate beyond established models to comprehensively elucidate EPEC pathogenesis.

## Introduction

Enteropathogenic *Escherichia coli* (EPEC) strains constitute one of the primary causes of severe and sometimes fatal childhood diarrhoea in developing countries [1,2]. Following infection, EPEC intimately attaches to the cell surface, inducing a lesion characterized by the loss of microvilli at the enterocyte brush border, cytoskeletal rearrangements and actin polymerization, culminating in the formation of pedestal-like structures at the bacterial attachment site, a defining feature known as the attaching and effacing (A/E) lesion [3]. The genes essential for A/E lesion formation reside within a horizontally acquired pathogenicity island spanning 35–43 kb, termed the locus of enterocyte effacement (LEE) [4]. The presence of the LEE distinguishes a group of pathogenic *E. coli* known as A/E pathogens, which includes, in addition to EPEC, Shiga toxin-producing enterohaemorrhagic *E. coli*, the mouse pathogen *Citrobacter rodentium*, rabbit enteropathogenic *E. coli* and *E. albertii* [5,7].

The LEE encodes the components of a type III secretion system (T3SS), a molecular syringe that translocates a myriad of effector proteins into the host cell. The genes within the LEE region are organized into five polycistronic operons (*LEE1–LEE5*), two bicistronic operons (*LEE6* and *LEE7*), and four transcriptional units (*etgA, cesF, map* and *escD*). The *LEE1*, *LEE2* and *LEE3* operons encode proteins that are components of the T3SS, including the *esc* and *sep* genes. *LEE4* contains genes encoding the EspA, EspB and EspD proteins, which form the translocon. *LEE5* includes genes encoding proteins essential for intimate adherence: Tir, CesT and intimin [4].

In addition, the presence or absence of the EPEC adherence factor plasmid (pEAF), which carries the *bfp* operon encoding the bundle-forming pilus (BFP) responsible for the localized adherence (LA) phenotype, divides EPEC strains into two main categories: typical EPEC (tEPEC) and atypical EPEC (aEPEC), respectively [8]. Although aEPEC strains lack the *bfp* operon present in typical strains, they possess alternative virulence factors that contribute to their pathogenicity [9]. While aEPEC does not induce the LA phenotype characteristic of tEPEC, it still adheres to intestinal cells and causes gastrointestinal symptoms. Infections caused by aEPEC are generally associated with milder forms of diarrhoea and affect a wider age range, including older children and adults [10,11]. Despite differences in adherence mechanisms and pathogenesis, both tEPEC and aEPEC strains pose significant public health risks due to their capacity to cause gastrointestinal infections.

The proper assembly of the T3SS depends on the coordinated and timely expression of LEE genes in response to various environmental signals [4,12, 13]. Several studies have demonstrated that Ler (LEE-encoded regulator), a 15 kDa protein encoded by the first gene of the *LEE1* operon, serves as the central positive regulator of LEE gene expression by counteracting H-NS repression at LEE promoters [14,17]. The regulation of Ler expression, and consequently of the LEE, involves a complex interplay of both positive and negative transcriptional regulators, as well as post-transcriptional events [5,13, 18,20].

The specific positive regulation of *ler* is mediated by two distinct horizontally acquired regulators: GrlA (global regulator of LEE-activator) and PerC (plasmid-encoded regulator), encoded within the LEE and pEAF plasmid, respectively [14,15, 21,29]. In contrast, GrlR (global regulator of LEE-repressor), also encoded in the LEE, acts as a negative regulator [30,32].

Ler further regulates GrlA expression, establishing a feedforward positive regulatory loop that ensures the production of the appropriate level of Ler protein to activate LEE genes [14]. GrlR forms a homodimer that suppresses LEE expression by binding to the HTH domain of GrlA, forming a heterotrimeric structure that prevents GrlA from interacting with the *LEE1* promoter [23,26, 30, 32,34]. Moreover, GrlR represses LEE gene expression through an additional mechanism that has yet to be fully elucidated [30]. In tEPEC, *ler* expression can be activated independently through two distinct mechanisms involving PerC and GrlA, depending on the growth conditions, either in Dulbecco’s Modified Eagle Medium (DMEM) static cultures with 5% CO_2_ or in shaken cultures, respectively [35].

Research on tEPEC regulatory mechanisms has primarily focused on the prototype strain E2348/69 (O127:H6). However, previous studies have highlighted the genetic heterogeneity within EPEC strains [36,44], though the impact of this diversity on LEE regulation remains largely unknown.

Historically, LEE gene regulation has been studied under *in vitro* conditions that either induce, such as growth in DMEM, or repress expression, such as growth in lysogeny broth or Luria Broth (LB). Protein secretion assays provide a functional assessment of LEE gene expression or T3SS activity under different experimental conditions. EPEC secretes the translocator proteins EspA, EspB and EspD [15,35, 45], making this assay a key indicator of T3SS activation and, consequently, functionality.

Despite advances in understanding aEPEC and tEPEC pathogenesis, the variability in LEE gene regulation among clinical isolates remains insufficiently explored. This study addresses this gap by analysing secretion profiles of a collection of clinical isolates under growth conditions that favour either PerC- or GrlA-mediated LEE expression. We analysed the type III secretion (T3S) profiles of 181 tEPEC and aEPEC isolates from children under 2 years of age, categorizing them into four distinct phenotypes based on their protein secretion profiles. Our findings revealed significant variability in secretion phenotypes that did not correlate with the clinical manifestations observed in the hosts. From this analysis, we selected two tEPEC strains exhibiting non-secretory and unregulated secretory phenotypes across all tested conditions for further investigation.

## Methods

### Bacterial strains, plasmids and growth conditions

The bacterial strains and plasmids used in this study are described in Table 1. We employed a collection of 181 strains, which were classified as tEPEC or aEPEC based on the presence of the *ler* (LEE) and/or *bfpA* genes. These strains were isolated during a previous passive surveillance cohort study involving 1034 children aged 2–24 months. Children were enrolled in the study starting at 2 months of age. Participants were required to reside in low-income communities within the peri-urban districts of Lima. Written informed consent was obtained from the parents prior to enrollment. At the time of control sample collection, the children had to be in good health, with no episodes of diarrhoea at least 1 week before or after the sample was taken [46]. Bacterial strains were cultivated in LB broth or DMEM containing glucose (0.45% [wt/vol]) and L glutamine (584 mg l^−1^), but not sodium pyruvate (GIBCO, Invitrogen corporation). When required, antibiotics were added at the following concentrations: ampicillin, 100 µg ml^−1^; streptomycin, 100 µg ml^−1^; kanamycin, 25 µg ml^−1^; and tetracycline, 10 µg ml^−1^.

**Table 1.** Bacterial strains and plasmids

Strain or plasmid	Description	Source
**EPEC**		
E2348/69	Prototype EPEC O127:H6, Sp^R^	[81]
JPEP22	E2348/69 derivative, ∆*perC*::kan	[35]
JPEP24	E2348/69 derivative, ∆*ler*::kan	[35]
JPEP25	E2348/69 derivative, ∆*grlA*::kan	[35]
JPEP26	JPEP22 derivative, ∆*perC*::kan ∆*grlA*	[35]
JPEP46	E2348/69 derivative, ∆*grlR*::kan	A. Vazquez
JPEP47	E2348/69 derivative, ∆*grlRA*::kan	A. Vazquez
JPEP48	JPEP22 derivative, ∆*perC*	This study
JPEP49	JPEP24 derivative, ∆*ler*	This study
JPEP50	JPEP25 derivative, ∆*grlA*	This study
JPEP51	JPEP22 derivative, ∆*grlR*	This study
JPEP52	JPEP47 derivative, ∆*grlRA*	This study
JPEP53	JPEP26 derivative, ∆*perC* ∆*grlA*	This study
D3158	Group 1 tEPEC clinical isolate, Amp^R^, Tet^R^	[46]
JPEP54	D3158 derivative, ∆*ler*::kan	This study
JPEP55	D3158 derivative, ∆*grlA*::kan	This study
JPEP56	D3158 derivative, ∆*grlR*::kan	This study
JPEP57	D3158 derivative, ∆*grlRA*::kan	This study
JPEP58	D3158 derivative, ∆*perC*::kan	This study
JPEP59	JPEP54 derivative, ∆*ler*	This study
JPEP60	JPEP55 derivative, ∆*grlA*	This study
JPEP61	JPEP60 derivative, ∆*perC*::kan ∆*grlA*	This study
JPEP62	JPEP61 derivative, ∆*perC* ∆*grlA*	This study
JPEP63	JPEP56 derivative, ∆*grlR*	This study
JPEP64	JPEP57 derivative, ∆*grlRA*	This study
D3309	Group 3 tEPEC clinical isolate, Amp^R^, Tet^R^	[46]
JPEP65	D3309 derivative, ∆*ler*::kan	This study
JPEP66	D3309 derivative, ∆*grlA*::kan	This study
JPEP67	D3309 derivative, ∆*grlR*::kan	This study
JPEP68	JPEP65 derivative, ∆*ler*	This study
JPEP69	JPEP66 derivative, ∆*grlA*	This study
JPEP70	JPEP67 derivative, ∆*grlR*	This study
***E. coli* K12**		
DH5α	Laboratory cloning strain	[82]
**Plasmids**		
pMPM-K3	Low-copy-number cloning vector, p15A derivative, Kan^R^	[83]
pKEP-PerC	pMPM-K3 derivative expressing *perC* from the *lac* promotor	This study
pKEP-Ler	pMPM-K3 derivative expressing *ler* from the *lac* promoter	This study
pKEP-GrlA	pMPM-K3 derivative expressing *grlA* from the *lac* promoter	This study
pKEP-GrlR_E2348_	pMPM-K3 derivative expressing *grlR* from the *lac* promoter	This study
pKEP-GrlR_D3158_	pMPM-K3 derivative expressing *grlR*_D3158_ from the lac promoter	This study
pKD46	Red recombinase system under control of *araB* promoter; Ap^R^	[50]
pKD4	Template plasmid containing the Kan cassette for lambda Red recombination	[50]
pCP20	Temperature-sensitive plasmid for Flp-catalysed excision of antibiotic-resistance cassette. Ap^R^ Cm^R^	[50]
psepZ-11	*cat* transcriptional fusion containing the *LEE2* promoter region	[15]

Both shaken and static + CO_2_ cultures were initiated by inoculating 5 ml of LB or DMEM containing 1% LB in 15 ml tubes with a 50-fold dilution of bacterial overnight cultures that were subsequently incubated in a shaken air incubator at 200 r.p.m. or without agitation in a CO_2_ incubator at 37 °C. In the case of static + CO_2_ cultures, prior to inoculation, the DMEM-containing tubes underwent pre-equilibration overnight in a 5% CO_2_ atmosphere at 37 °C. We collected samples for protein secretion and CAT assays and Western blot analysis when the cultures reached an OD_600_ of approximately 1, which corresponded to approximately 6, 7 and 8 h of growth for DMEM and LB shaken and static + CO_2_ cultures, respectively.

### Protein secretion assay

EPEC secreted proteins were analysed as previously described [35]. In brief, 5 ml of DMEM or LB medium was inoculated with 50 µl of an overnight LB culture of the selected strains. Cultures were incubated at 37 °C under two conditions: shaking at 200 r.p.m. and static + CO_2_ until reaching an OD_600_ of ~1. Bacteria were centrifuged for 5 min at 17 900 *g*, and the supernatant was collected. Secreted proteins were precipitated with 10% trichloroacetic acid (TCA) at 4 °C overnight and concentrated by centrifugation at 17 900 *g* for 30 min at room temperature. The precipitated proteins were resuspended in loading buffer (47.6% glycerol, 4% sodium dodecyl sulfate (SDS), 0.1% β-mercaptoethanol, 0.24% Tris-HCl pH 6.8 and 0.02% bromophenol blue), and 1 µl of 1.0 M Tris pH 11 was added to neutralize the TCA. The samples were boiled for 5 min, subsequently resolved by 15% SDS-polyacrylamide gel electrophoresis (PAGE) and visualized by Coomassie brilliant blue R-250 staining (Bio-Rad).

### Western blotting

To prepare whole-cell extracts, bacterial pellets from culture samples were resuspended in 210 µl of 3M urea at pH 8.0 and 70 µl of 4X Laemmli sample buffer. The proteins were separated by 15% SDS-PA GE and transferred onto a nitrocellulose membrane (Millipore) using a semidry transfer apparatus (Bio-Rad). Subsequently, the membranes were blocked with 5% nonfat milk, washed with PBS-T and incubated with a 1 : 50  000 dilution of rabbit polyclonal anti-GroEL (Sigma-Aldrich), a 1 : 10 000 dilution of rabbit polyclonal anti-EscJ or 1 : 1000 dilution of rabbit polyclonal anti-GrlR. The membranes were washed again, then incubated with a 1 : 10 000 dilution of horseradish peroxidase-conjugated anti-rabbit antibody (Rockland) and developed using the Western Lightning Plus-Enhanced Chemiluminescent Substrate (Perkin-Elmer) following the manufacturer’s instructions. Bands were detected using X-OMAT LS films (Carestream, Sigma).

### Chloramphenicol acetyltransferase (CAT) assay

CAT activity was assessed following the methodology previously described [47,48]. Briefly, strains carrying the *sepZ11-cat* transcriptional fusion, which contains the promoter region of the *LEE2* operon, activated directly by Ler and indirectly by GrlA and PerC in response to the growth conditions [35], were cultured in LB or DMEM at 37 °C under shaking or static with CO_2_ conditions until reaching an OD_600_ of 0.8–1.0. Samples were collected, and cells were pelleted, washed with TDTT buffer (50 mM Tris-HCl pH 7.8, 30 μM dl-dithiothreitol) and lysed by sonication. Soluble extracts were prepared and added to a microtiter plate. Changes in absorbance at 410 nm were monitored for 5 min after adding a reaction mixture containing 1 mM DTNB [5,5'-dithiobis (2-nitrobenzoic acid)], 0.1 mM acetyl-coenzyme A (acetyl-CoA), and 0.1 mM chloramphenicol in 0.1 M Tris-HCl, pH 7.8. CAT activity was determined using a standard curve (expressed in U ml^−1^), and protein concentration was measured using the BCA Protein Assay Kit. CAT-specific activity (expressed as µmol/min/mg of protein) was then calculated. Results were derived from three independent assays, each performed in duplicate. Minimal CAT activity was observed in strains with the empty vector pKK232-8.

### DNA manipulations

DNA manipulations were performed according to standard protocols [49]. Restriction enzymes were obtained from Thermo Scientific and used according to the manufacturer’s instructions. The oligonucleotides used in this work are listed in Table 2 and were synthesized at the oligonucleotide synthesis facility of the Instituto de Biotecnología/UNAM.

**Table 2.** Primers used in cloning and mutagenesis experiments

Primer	Sequence (5′ to 3′)*	Target gene
**For gene cloning**		
EPGA-XI (fw)	gccaaatttcTCGAGccattaatta	*grlA*_E2348_
EpCiorf11R (rv)	tactaagaAAGCTtcgtctaactctcc	
plerEPBH-F (fw)	taaggatCCggtcgctaatagc	*ler*_E2348_
plerEPSI-R (rv)	cttcGagctcagttatcgttatc	
BFPW-HIND (rv)	actcaaGctTcgttataatttaa	*perC*_E2348_
BFPW-NCO (fw)	ggtaaCCATGGaaataagagat	
EPgrlR-Hind (rv)	tgataaataaaGcTTcataa	*grlR*_E2348_
EPgrlR-KpnI (fw)	cgttgGtacCcaatattattaatc	
grlR-34-Fw (fw)	ggcGGTACctcaatattattaatcag	*grlR*_D3158_
grR-34-Rv (rv)	cgggcAaGCtTgacataaaaaacatgcat	
**For gene deletion**		
EgrlR-H1P1 (fw)	tatgaaactgagtgagttatgattatgaaggatggcatctatagctgtaggctggagctgcttcg	*grlR*
EgrlR-H2P2 (rv)	gtattttttgtatgtatttttaataagatttatttgaacaccgtacatatgaatatcctccttag	
Eler-H1P1 (fw)	aatagcttaaaatattaaagcatgcggagattatttattatgaattgtaggctggagctgcttcg	*ler*
Eler-H2P2 (rv)	tcatttaattatttcatgttaaatatttttcagcgctattatttccatatgaatatcctccttag	
EgrlA-H1P1 (fw)	ataaaaagaatatggaaaatggaatctaaaaataaaaatggcgactgtaggctggagctgcttcg	*grlA*
EgrlA-H2P2 (rv)	aatatactcaaaaaattacgtctaactctcctttttccgcctcaacatatgaatatcctccttag	
EP34-grlR-H1P2 (fw)	tatgaaactgagtgagttatgattatgaaggatggcatctatagccatatgaatatcctccttag	*grlR*
EP34-grlR-H2P1 (rv)	cataaaaaacatgcataaaaattattttaaataaacttatggcattgtaggctggagctgcttcg	
**For RT-PCR**		
EPEC-gyrB-RT-F (fw)	accattcacgccgataactc	*gyrB*
EPEC-gyrB-RT-R (rv)	gggcgtttactaccgaaaca	
EPEC- ler-RT-F (fw)	gactgcgagagcaggaagtt	*ler*
EPEC- ler-RT-R (rv)	caggtctgcccttcttcatt	
EPEC- grlA-RT-F (fw)	gccgaagcattcggtataaa	*grlA*
EPEC- grlA-RT-R (rv)	ttttctttttggtccggttg	
EPEC- grlR-RT-F (fw)	aagactcctgtggggaaggt	*grlR*
EPEC- grlR-RT-R (rv)	aatgtttagcaccgagggaat	
EPEC- escR-RT-F (fw)	ctgttaccggctttcacgat	*escR*
EPEC- escR-RT-R (rv)	ataatttttgccagcctcca	
EPEC- cesD-RT-F (fw)	atctctgggcttatgccatc	*cesD*
EPEC- cesD-RT-R (rv)	aaggctttcttggccatttt	
EPEC- escJ-RT-F (fw)	gcaagcactgttgctatcca	*escJ*
EPEC- escJ-RT-R (rv)	gctgggtgggaaaataacct	
EPEC- mpc-RT-F (fw)	ctttagtgcccttgctcctg	*cesL*
EPEC- mpc-RT-R (rv)	gaacgcgctcaataatctgc	
EPEC- of15-RT-F (fw)	ttgcctctaagcaggcgtat	*escO*
EPEC- of15-RT-R (rv)	gcaatgaacgcttttccttc	
EPEC- speL-RT-F (fw)	caaaggtagcgcaaggaaag	*sepL*
EPEC- speL-RT-R (rv)	atcgccaaagtaggatcgtg	
EPEC- espB-RT-F (fw)	aggctcttttgctgccatta	*espB*
EPEC- espB-RT-R (rv)	tctgctgcatctgcaatacc	
EPEC-espZ-RT-F (fw)	ttaagcccttctggtgcagt	*sepZ*
EPEC-espZ-RT-R (rv)	tttgtgaagggtcgtcaaca	
EPEC-tir-RT-F (fw)	caaattggaccctctgcatt	*tir*

*Capital letters indicate changes designed to introduce restriction sites. The underlined sequence corresponds to the template plasmids pKD4 or pSUB11. ‘fw’ denotes forward, and ‘rv’ denotes reverse.

### Construction of plasmids

To construct expression plasmids for *ler*, *grlA*, *grlR* and *perC* of the EPEC strain E2348/69, we amplified PCR fragments containing the respective structural genes using primers pairs plerEPBH-F/plerEPSI-R, EPGA-XI/EpCiorf11R, EPgrlR-Hind/EPgrlR-KpnI and BFPW-HIND/BFPW-NCO, with EPEC E2348/69 chromosomal DNA as the template. The fragments containing *ler*, *grlA*, *grlR* and *perC* were digested with BamHI/PstI, XhoI/HindIII, HindIII/KpnI and HindIII/NcoI, respectively, and then cloned into vector pMPM-K3 digested with the same combinations of enzymes. For the construction of plasmid pKEP-GrlR_D3158_, a PCR fragment containing the *grlR* structural gene was obtained using primers grlR-34-Fw and grlR-34-Rv with EPEC D3158 chromosomal DNA as the template. This fragment was then digested with KpnI and HindIII before being cloned into pMPM-K3, which was also digested with the same enzymes.

### Construction of EPEC deletion mutants

To generate EPEC non-polar gene deletion mutants, we used the one-step method based on the lambda Red recombinase system [50] encoded in plasmid pKD46 (Table 1). The PCR fragments used to delete and replace the *ler, perC, grlA* and *grlR* genes with a kanamycin cassette were amplified using oligonucleotides pairs Eler-H1P1/Eler-H2P, EperC-H1P1/EperC-H2P2, EgrlA-H1P1/EgrlA-H2P2 and EgrlR-H1P1/EgrlR-H2P2, respectively (Table 2), and plasmid pKD4 (Table 1) DNA as template. The resistance cassette was excised by introducing the temperature-sensitive plasmid pCP20 (Table 1) that expresses the Flp recombinase.

### RNA purification and qRT-PCR

Bacterial strains were grown in 50 ml of DMEM shaken, DMEM static + CO_2_ and LB at 37 °C until reaching an OD_600_ of ∼0.6, after which 80 µl of rifampicin (125 µg ml^−1^) was added. Cellular pellets were collected by centrifugation at 10 000 r.p.m. for 15 min at 4 °C and washed twice with diethyl pyrocarbonate (DEPC)-treated water and then stored at −70 °C. Total RNA was extracted from the stored pellets as follows. To each pellet, 400 µl of SET buffer (10 mM Tris pH 7.5, 10 mM EDTA and 0.5% SDS) was added and gently mixed until fully suspended. Subsequently, 400 µl of phenol acid solution pH 4 (Invitrogen, Van Allen Way, USA) was added, and the mixture was incubated for 45 min at 65 °C with agitation (600 r.p.m.), followed by 5 min on ice. Then, it was centrifuged at 12 000 *g* for 15 min at room temperature. This step was repeated, and the aqueous phase was recovered. To the recovered phase, 300 µl of phenol and 300 µl of chloroform–isoamyl alcohol (1 : 1) were added, mixed by inversion for 30 s and centrifuged at 12 000 *g* for 15 min at room temperature. The last extraction was performed with 500 µl of chloroform. To precipitate the solution, 60 µl of 3 M sodium acetate pH 5.2, 1 ml of 100% ethanol (J.T.Baker) and 1.3 µl of 500 mM EDTA were added, mixed by inversion and incubated at −70 °C for 30 min. To obtain the RNA pellet, the mixture was centrifuged at 14 000 *g* for 25 min at 4 °C. The supernatant was discarded, and the pellet was washed with 1 ml of 70% ethanol diluted in DEPC-treated water, followed by centrifugation at 14 000 *g* for 5 min at 4 °C. The pellets were vacuum-dried for 25 min without heat. The RNA pellet was resuspended in 50 µl of DEPC-treated water and stored at −70 °C in 15 µl aliquots. RNA was analysed on 2% agarose gels (Axigen) and quantified using NanoDrop 2000c (Thermo Scientific, Wilmington, USA) at absorbances of 260 and 280 nm. To ensure that the RNA sample was free from genomic DNA, RNA samples were treated with the commercial DNase I kit (Fermentas, Burlington, Canada) following the manufacturer’s recommendations.

Quantitative real-time PCR (qRT-PCR) was performed as follows. The RevertAid H minus First Strand cDNA Synthesis kit (Thermo Scientific) was used according to the manufacturer’s specifications to produce cDNA from the different RNA samples using 2 µM of oligonucleotides lerRT-Rv, grlART-Rv, perCRT-Rv, grlRRT-Rv and gyrART-Rv (Table 2). qRT-PCR reactions were carried out in a final volume of 15 µl, with a mixture of 7.5 µl of Maxima SYBR Green/ROX qPCR Master Mix (2X) (Thermo Scientific), 0.5 µl of each 20 mM oligonucleotide and 1 µl of cDNA (5 ng µl^−1^). The mix was run in a LightCycler 480 Instrument (Roche Diagnostics). Data were collected using LightCycler 480 software, normalized to endogenous *gyrB* levels and analysed using the comparative critical threshold method. qRT-PCR experiments were performed in two independent experiments. All data were expressed as means±SD from triplicate analysis. Statistical significance was determined by a Student’s *t*-test based on comparisons with strain E2348/69.

## Results

### T3SS-mediated secretion exhibits variable phenotypes in EPEC clinical isolates

The expression of the LEE of EPEC E2348/69 is modulated in response to the growth conditions and by a broad network of transcriptional regulators, mainly acting on the expression of the master regulator Ler [13]. For instance, when EPEC grows in rich medium such as LB, the LEE is expressed poorly, whereas in DMEM, its expression increases significantly compared with LB [35,51]. GrlA and PerC, two independent activators of *ler* expression, each respond to distinct growth conditions. GrlA predominantly exerts its influence during EPEC E2348/69 growth in shaken DMEM, whereas PerC’s impact is more pronounced in static DMEM cultures under a 5% CO_2_ atmosphere [35]. Building on this knowledge, we set out to investigate whether this dynamic phenotype, associated with the two specific *ler* activators, GrlA and PerC, is conserved in clinical isolates of tEPEC strains that possess the EAF plasmid and, therefore, the *perC* gene, as well as in isolates of aEPEC which, by definition, lack pEAF and *perC*.

Considering that the type III protein secretion profile of EPEC, characterized mainly by the translocator proteins EspB, EspD and EspA, serves as an indicator of the activation status and functionality of the LEE, we conducted a qualitative analysis of secretion profiles among 43 tEPEC strains and 138 aEPEC strains from a prior cohort study in Peru [46]. These strains were cultured in both shaken DMEM and static DMEM under a 5% CO_2_ atmosphere at 37 °C.

**Fig. 1.**
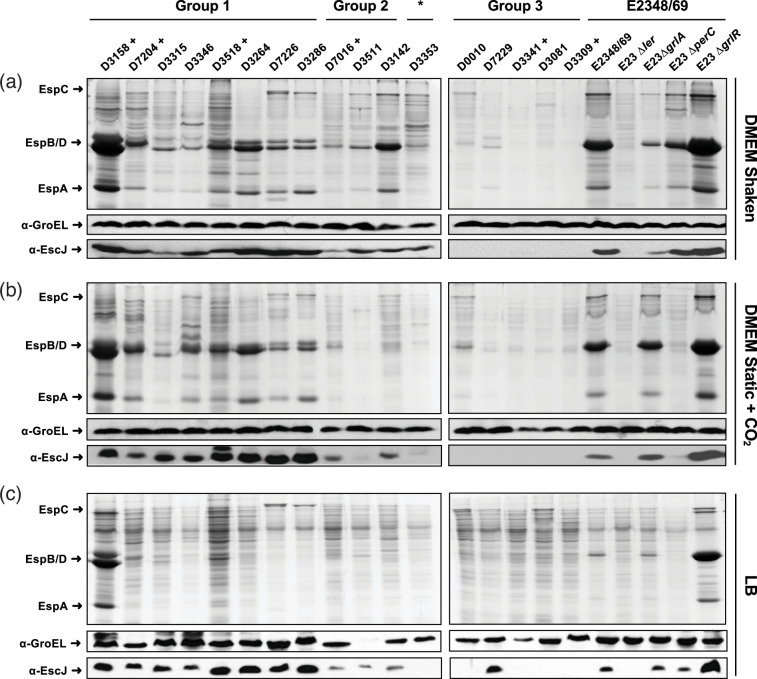
The protein secretion profiles of 17 representative tEPEC and aEPEC clinical isolates demonstrate the diversity within EPEC. Bacteria were cultured under various conditions: shaken DMEM (**a**), static DMEM in a 5% CO_2_ atmosphere (**b**), and shaken LB medium (**c**), all at 37 °C. Secreted proteins were precipitated from culture supernatants collected at the stationary phase using 10% TCA and resolved by 15% SDS-PAGE. Total cell extracts were subjected to Western blot analysis using anti-EscJ (*LEE2*) and anti-GroEL antibodies as loading controls. The strains were categorized into three groups: Group 1 (lanes 1 to 8), Group 2 (lanes 9 to 11), and Group 3 (lanes 12* to 17). The experiment was replicated at least three times, yielding similar results. The symbol ‘+’ indicates tEPEC strains carrying the pEAF plasmid.

Based on their observed protein secretion patterns, the 181 EPEC isolates were categorized into four distinct groups (Table 3): Group 1 comprised strains consistently showing secretion under both growth conditions; Group 2 encompassed strains primarily exhibiting secretion in shaken DMEM; Group 3 consisted of strains that, across at least three separate assays, showed poor or no detectable levels of secreted proteins under either culture condition (Fig. 1). The group labelled NR included three tEPEC strains and 13 aEPEC strains that, in various assays, did not exhibit a consistent secretion profile or displayed tendencies toward cell lysis, so they were not included in any of the groups.

**Table 3.** Classification of EPEC strains based on their protein secretion profiles

	tEPEC(*n* = 43) (%)	aEPEC(*n* = 138) (%)	All strains(*n* = 181) (%)
Group 1	30 (69.8)	56 (40.6)	86 (47.5)
Group 2	1 (2.4)	11 (7.9)	12 (6.6)
Group 3	9 (20.9)	58 (42.1)	67 (37.1)
NR	3 (6.9)	13 (9.4.)	16 (8.8)

Remarkably, among the 138 aEPEC strains lacking the EAF plasmid, and therefore *perC*, 56 (40.6%) (Group 1) exhibited secretion of translocator proteins under both growth conditions, albeit to varying extents. Conversely, among the 43 tEPEC strains, 30 (69.8%) (Group 1) displayed behaviour reminiscent of the prototype strain by secreting under both conditions. In contrast, 58 (42.1%) of the aEPEC strains and 9 (20.9%) of the tEPEC strains (Group 3) showed negligible secretion levels under the tested conditions, including shaken DMEM, where E2348/69 Wt, Δ*perC* or pEAF-cured strains secrete. Notably, only a minority of aEPEC strains, specifically 11 (7.9%) out of 138 and 1 (2.4%) out of 43 tEPEC strains (Group 2), secreted solely in shaken DMEM, closely resembling the phenotype of the E2348/69 Δ*perC* strain (Table 3).

For the subsequent phase of the study, 17 strains were selected to reflect the secretion phenotypic diversity observed across the 3 groups. Group 1, which exhibited translocator protein secretion under both growth conditions, is represented by eight strains (five aEPEC and three tEPEC strains). From Group 2, three strains were chosen (two aEPEC and one tEPEC), characterized by secretion only in shaken DMEM, resembling the E2348/69 Δ*perC* strain. Finally, six strains were chosen from Group 3, which showed negligible secretion under the tested conditions, including four aEPEC and two tEPEC strains. This selection provided a broad representation of the secretion phenotypes, enabling further exploration of the regulatory mechanisms involved in these distinct secretion patterns.

The selected strains were grown in DMEM under shaken and static + CO_2_ conditions, as well as in LB medium. In addition to assessing secretion profiles by collecting supernatants, cell pellet samples were obtained to quantify EscJ levels, a structural, non-secreted component of the T3SS, using Western blotting. This approach provided an additional indirect measure of LEE expression levels. As shown in Fig. 1, the overall expression of EscJ correlates with both the secretion profile and the group assignment of each strain.

These findings demonstrated considerable variability and heterogeneity in T3S among EPEC strains, particularly in aEPEC strains. Notably, this variability did not correlate with the presence or absence of the pEAF plasmid, thereby hindering any association with the clinical presentation associated with each isolate (Table 4).

**Table 4.** Clinical information and phenotypes of the 17 EPEC clinical strains selected

Strain information				Clinical information		Phenotype
**Strain**	O type*	**H type†**	**tEPEC/ aEPEC‡**	**Patient age§**	**Diarhoea/control¶**	Secretion profile**
D3158	2w	+	T	7.8	D	G1
D7204	119 w	6 or 41	T	6.8	D	G1
D3315	−	33	A	19.9	D	G1
D3346	116	33	A	20.2	D	G1
D3518	−	−	T	5.4	D	G1
D3264	128	2	A	5.1	D	G1
D7226	119	8	A	4.5	D	G1
D3286	−	8	A	20.3	C	G1
D7016	−	+	T	5.7	D	G2
D3511	−	10	A	11.6	C	G2
D3142	−	10	A	8.1	D	G2
D3353	−	10	A	15.2	C	G3
D0010	−	8	A	9.1	D	G3
D7229	−	11	A	11.1	C	G3
D3341	55	21 or 36	T	7.6	D	G3
D3081	153	19	A	12.3	C	G3
D3309	−	5	T	9.8	C	G3

*Determined by serology.

†Determined by PCR.

‡tEPEC and aEPEC were distinguished based on the presence or not of *bfp*A.

§Children’s age in months.

¶Strains isolated from diarrhoea (D) and control (without diarrhoea) (C) samples.

**According to Fig. 1 and Table 3.

### GrlA or PerC expression, or GrlR elimination, overcomes LEE expression silencing in a non-secretory Group 3 tEPEC strain

Given that the assembly of the T3SS requires the coordinated and proper expression of all LEE operons [4,12], we investigated whether the lack of secretion observed in Group 3 tEPEC strains under inducing conditions could be attributed to mutations in the *ler*, *grlA* or *perC* regulatory genes, which may either impair their expression or result in non-functional proteins. Alternatively, we considered the possibility that additional factors could be reinforcing LEE repression, which may be overcome under specific environmental conditions different from those documented for EPEC E2348/69.

To explore these possibilities, we examined tEPEC strain D3309 (Table 4), which neither secreted translocator proteins nor expressed EscJ under inducing conditions (Fig. 1a). Moreover, this strain showed markedly lower levels of LEE gene expression compared to the prototype strain, as determined by RT-PCR (Fig. S1, available in the online Supplementary Material). Analysis of the draft genome sequence of tEPEC strain D3309 revealed that the genes encoding Ler, GrlA, PerC and GrlR, along with their respective regulatory regions, are almost identical to those of the E2348/69 prototype strain (unpublished results), suggesting that their likely expressed and produce functional protein.

**Fig. 2.**
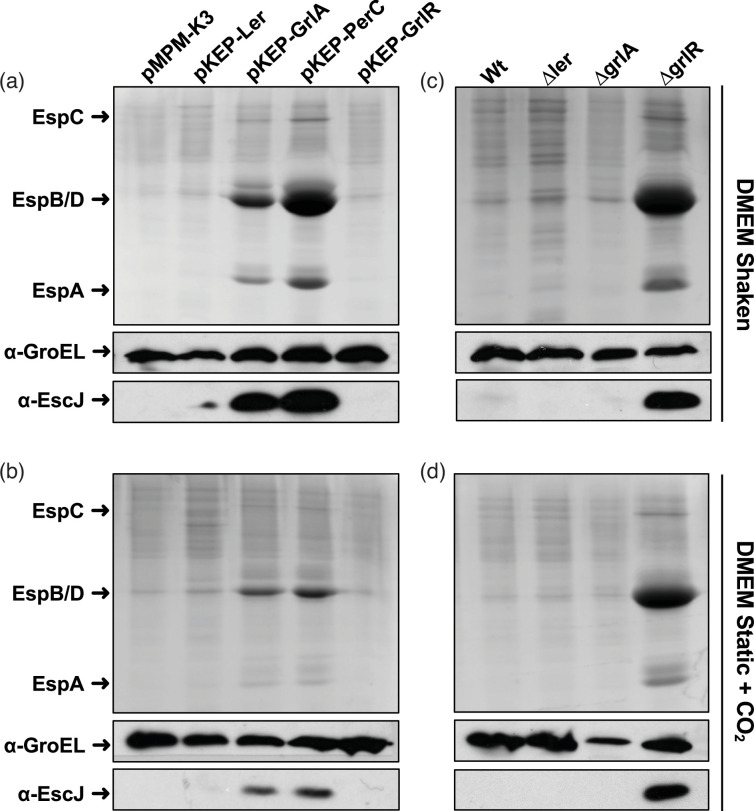
Constitutive expression of GrlA and PerC and elimination of GrlR restored T3S and LEE expression in the non-secreting tEPEC strain D3309. tEPEC strain D3309 transformants carrying plasmids pMPM-K3 (empty vector), pK3-Ler_E2348_, pK3-GrlA_E2348_, pK3-PerC_E2348_ or pK3-GrlR_E2348_ (a and b) or WT and its Δ*ler*, Δ*grlA* and Δ*grlR* isogenic mutants (c and d) were grown in shaken DMEM or static DMEM + CO_2_. Culture supernatants and bacterial pellets were collected at an OD_600_ of approximately 1.0. Secreted proteins were precipitated using 10% TCA, resolved by 15% SDS-PAGE and stained with Coomassie brilliant blue. Western blot analysis of total cell extracts from the same strains was conducted using α-GroEL as a loading control (middle panels) and α-EscJ to detect the non-secreted LEE-encoded T3SS component (bottom panels). The experiment was replicated at least three times, yielding similar results.

Subsequently, we introduced plasmids carrying the genes encoding Ler, GrlA, PerC and GrlR regulatory proteins from the prototype tEPEC strain E2348/69 into strain D3309 (Table 1). The resultant strains were cultured under inducing conditions (shaken DMEM or static DMEM + CO_2_) to assess the impact of regulators’ overexpression on LEE gene expression. Notably, the presence of GrlA and PerC restored of EspB/D and EspA secretion, as well as EscJ expression, to detectable levels, as confirmed by Coomassie blue staining or Western blot analysis, respectively (Fig. 2a, b). This suggests that strain D3309 possesses a functional secretion system and that the observed lack of secretion is likely due to limited Ler expression. Consistent with this interpretation, deletion of the *grlR* gene in tEPEC D3309 resulted in a significant increase in translocator protein secretion (Fig. 2c, d) and, presumably, LEE gene transcription, as observed for the *grlR* mutant of the prototype tEPEC strain E2348/69 (Fig. 4b).

These data collectively indicate that, despite the high sequence conservation of LEE genes between the tEPEC strains E2348/69 and D3309, the mechanisms regulating their expression are influenced by factors that respond differently to environmental cues.

### Positive regulators PerC and GrlA are dispensable in a Group 1 tEPEC strain

We also investigated the intriguing phenotype of the tEPEC strain D3158, which notably secreted translocator proteins and expressed EscJ under all tested growth conditions, including LB (Fig. 1). As previously reported, PerC and GrlA independently activate *ler* expression, and consequently the LEE genes, in the prototype strain E2348/69 in response to different growth conditions, such as shaken DMEM and static DMEM + CO_2_ [35].

**Fig. 3.**
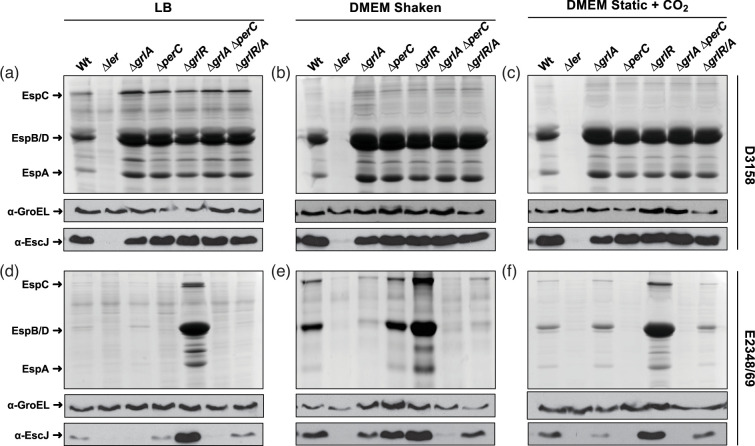
The secreted protein profile of the hypersecretory tEPEC D3158 strain is maintained even in the absence of GrlA and PerC. tEPEC strains D3158 and E2348/69 and their corresponding isogenic mutants, Δ*ler*, Δ*grlA*, Δ*perC*, Δ*grlR*, Δ*perC* Δ*grlA* and Δ*grlR/A*, were grown in LB medium with shaking (**a**), DMEM with shaking (**b**) and static DMEM under a 5% CO_2_ atmosphere (**c**), at 37 °C. Culture supernatants and bacterial pellets were collected at an OD_600_ of approximately 1.0. Secreted proteins were precipitated using 10% TCA, resolved by 15% SDS-PAGE and stained with Coomassie brilliant blue (upper panels). Western blot analysis of total cell extracts from the same strains using α-GroEL (loading control, middle panels) and α-EscJ (non-secreted LEE-encoded T3SS component, bottom panels). The experiment was replicated at least three times, yielding similar results.

Based on the above, we questioned whether GrlA and PerC play a similar role in the tEPEC strain D3158, and whether one of these regulators has a dominant role in LEE activation in LB medium, a condition that is repressive for the prototype strain. To investigate this, we generated mutant strains in the *ler*, *grlA*, *perC* and *grlR* regulatory genes, which are highly conserved between tEPEC strains D3158 and E2348/69, as well as *grlA/perC* and *grlRA* double mutants (Table 1). We then grew these strains under inducing (shaken DMEM and static DMEM + CO_2_) and repressing (LB) conditions, and compared their secreted protein profiles and EscJ expression (Fig. 3).

This analysis confirmed that, unlike the prototype strain, D3158 secretes even under repressive conditions. Interestingly, protein secretion and EscJ expression were abolished in the D3158∆*ler* mutant under all tested conditions, indicating that LEE expression in D3158 is also Ler-dependent. However, unexpectedly, the D3158∆*grlA* and D3158∆*perC* mutants, as well as the D3158∆*grlA*∆*perC* double mutant, showed secretion levels of EspB/D and EspA proteins and EscJ expression very similar to those of the wild-type strain, even in LB (Fig. 3a−c). In contrast, the E2348/69 strain and its mutants exhibited previously reported phenotypes [35], with the most striking being the E2348/69∆*grlA*∆*perC* strain, which displayed no LEE activity under any of the tested conditions.

**Fig. 4.**
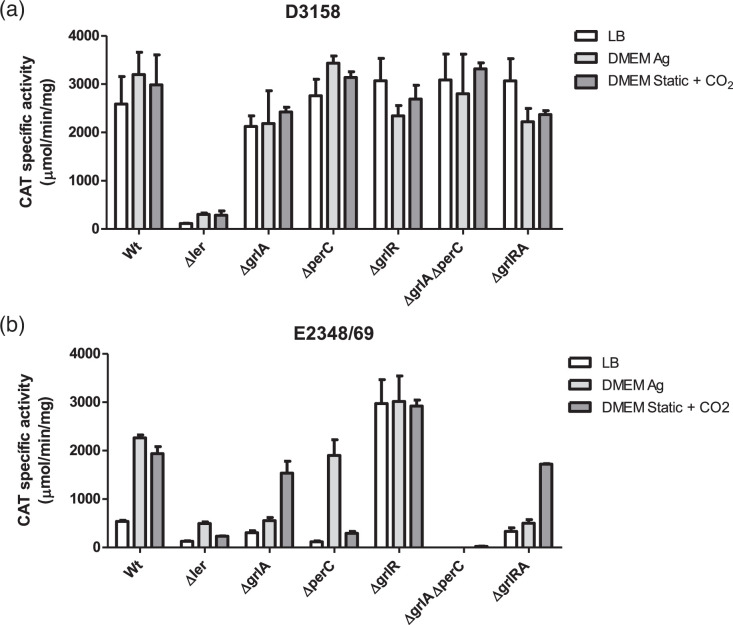
The transcriptional activity of the *sepZ11-cat* fusion in tEPEC D3158 remains active in the absence of the *ler*-specific activators GrlA and PerC, but it remains dependent on Ler. tEPEC strains D3158 (**a**) and E2348/69 (**b**), along with their corresponding isogenic mutants (Δ*ler*, Δ*grlA*, Δ*perC*, Δ*grlR*, Δ*perC*Δ*grlA* and Δ*grlR/A*), carrying a plasmid with the *sepZ11-cat* fusion, were cultured in LB medium with shaking, shaken DMEM and static DMEM under 5% CO_2_ at 37 °C. CAT-specific activity was determined from samples collected at an OD_600_ of ∼1. The data represent the average of three independent experiments conducted in duplicate.

We further validated these results by introducing plasmid psepZ-11 (*LEE2-cat* fusion), which contains the regulatory region of the *LEE2* operon fused to the promoterless *cat* reporter gene (Table 1), into these strains. The CAT activity determined from culture samples in the three media used in this study showed results consistent with the secretion profiles and EscJ expression described above (Fig. 3). Specifically, the absence of Ler in the D3158∆*ler* strain resulted in a significant reduction of fusion activity. In contrast, in the D3158∆*grlA*∆*perC* strain, CAT activity was comparable to that of the wild-type strain (Fig. 4a). As expected, the mutant strains derived from E2348/69 displayed the anticipated phenotypes (Fig. 4b).

### Constitutive LEE expression in the EPEC D3158 strain occurred despite having a functional GrlR variant

**Fig. 5.**
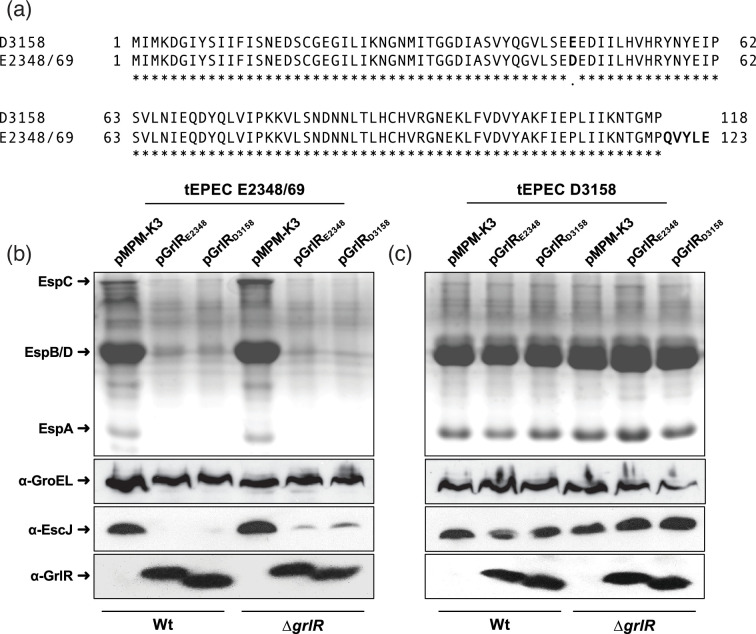
tEPEC strain D3158 expresses a functional GrlR protein that lacks the last five amino acids compared to that of strain E2348/69. Alignment of the amino acid sequences of GrlR from strains E2348/69 and D3158 (**a**). Secreted protein profiles of wild-type E2348/69 and D3158, along with their ∆*grlR* isogenic mutants, transformed with plasmids pMPM-K3 (empty vector), pK3-GrlR_E2348_ and pK3-GrlR_D3158_ (b and c, upper panels). Culture supernatants and bacterial pellets were collected at an OD_600_ of approximately 1.0. Secreted proteins were precipitated using 10% TCA, resolved by 15% SDS-PAGE and stained with Coomassie brilliant blue (upper panels). Western blot analysis of total cell extracts from the same strains using α-GroEL (loading control), α-EscJ (non-secreted LEE-encoded T3SS component) and α-GrlR antibodies (b and c, lower panels). The experiment was conducted three times independently, yielding similar results.

The regulatory and structural gene sequences of *ler* are identical between strains E2348/69 and D3158; thus, nucleotide sequence differences did not explain the apparent constitutive activity of the LEE_3158_ (data not shown). However, in the D3158 strain, the *grlR* gene encodes a protein lacking the last five amino acids compared with the E2348/69 strain (Fig. 5a).

We observed that overexpression of GrlR from a plasmid significantly reduced LEE gene expression in the wild-type E2348/69 strain, even under secretion conditions (Fig. 5b), but did not repress it in the D3158 strain. This finding was consistent with the clear derepression of the LEE observed in the E2348/69∆*grlR* mutant strain (Fig. 3d–f). In this context, we considered the possibility that the GrlR protein of the D3158 strain was either not expressed or non-functional and, therefore, led to constitutive LEE expression. To explore these options, we transformed wild-type E2348/69 and D3158, as well as their respective *grlR* mutants, with plasmids encoding GrlR_E2348_ or GrlR_D3158_. The resulting strains were grown under inducing conditions (shaken DMEM) to analyse their secretion profiles, and EscJ and GrlR expression as described above. The results showed a somewhat unexpected outcome, as while both GrlR_E2348_ and GrlR_D3158_ proteins repressed LEE expression in the E2348/69 strain, demonstrating that the last five amino acids are not essential for GrlR’s function as a repressor (Fig. 5b), neither of them was able to reduce the secretion of translocator proteins or EscJ expression in the D3158 strain, even though their expression was similar in both strains (Fig. 5c).

## Discussion

Significant progress has been made over the past two decades in understanding EPEC pathogenesis and regulation, largely through studies of prototype strains such as EPEC E2348/69 and B171 [13,19, 52, 53]. However, whole genome sequencing has revealed extensive genetic diversity among EPEC strains [39,41, 54,57], including complex hybrid pathotypes [38,58,62]. Therefore, relying solely on prototype strains may limit our comprehension of the full spectrum of EPEC pathogenesis and its regulatory mechanisms.

Two subtypes of EPEC are found in humans: tEPEC and aEPEC. While aEPEC comprises a heterogeneous group of strains that share the LEE pathogenicity island, it is considered an emerging pathogen [10,11, 41, 63, 64]. In contrast, tEPEC strains exhibit more homogeneous virulence traits, with studies showing a strong association between tEPEC infections and increased mortality in children under 5 years of age [57,65, 66]. Despite the association between tEPEC and morbidity and mortality in developing countries, it remains unclear how two strains with similar virulent genetic backgrounds cause distinct clinical outcomes.

In EPEC strain E2348/69, LEE expression is finely regulated by a balance between the repressor H-NS and its counteracting paralog, Ler [13,19]. The relative concentrations of these regulators fluctuate depending on growth conditions [51,67]. Ler expression is controlled by the positive regulators GrlA (LEE-encoded) and PerC (EAF plasmid-encoded in tEPEC), with their contributions varying according to the growth environment [35]. This dynamic regulatory interplay results in bimodal LEE gene expression during transitions between non-activating and activating conditions [51,67].

In this study, we observed substantial variability in LEE activation among clinical isolates of tEPEC and aEPEC, likely reflecting adaptations driven by distinct evolutionary pressures. A notable finding was that a significant proportion of aEPEC strains lacking the EAF plasmid, and consequently PerC, were still able to secrete translocator proteins under different growth conditions, contrasting with the secretion defects observed in Δ*perC* mutants of the prototype strain E2348/69. The absence of PerC has been proposed as a major factor hindering LEE expression and delaying the formation of A/E lesions in aEPEC strains [68]. However, the deletion of *grlR* in tEPEC D3309 led to increased translocator protein secretion, consistent with enhanced LEE gene transcription, as seen in the E2348/69 *grlR* mutant. These results suggest that aEPEC may employ alternative regulatory mechanisms that respond differently to environmental signals, reflecting their genomic diversity.

We also identified key differences in LEE regulation between strains D3158 and E2348/69. While LEE expression in D3158 remains dependent on Ler, this strain continues to secrete proteins under conditions typically repressive in E2348/69. Interestingly, deleting *grlA*, *perC*, or both does not significantly affect secretion or EscJ expression in D3158. This contrasts with the severe loss of LEE activity observed in similarly mutated E2348/69 strains, particularly the double mutant, which shows no activity under any condition. Additionally, despite missing the last five amino acids, the GrlR protein from D3158 effectively represses LEE expression in E2348/69. However, neither GrlR variant from E2348/69 nor D3158 reduce translocator protein secretion or EscJ expression in D3158. These findings suggest that D3158 uses an alternative mechanism to sustain Ler expression and LEE activity, even without GrlA and PerC, which are essential for *ler* activation in E2348/69 [28,35, 67]. This alternative mechanism will be a focus of future investigations.

Transcriptomic analyses of three prototype tEPEC strains (E2348/69, B171 and C581-05) revealed variable LEE gene expression patterns under both inducing and repressing growth conditions, as well as in response to the growth phase [54]. Notably, differential expression of *perC* was observed across these strains [54], and PerC plays a role in the bimodal expression of LEE in E2348/69 [67]. Such variability has also been noted in genetically diverse EPEC lineages [38,39, 69] and in virulence phenotypes [36,57, 63, 70], highlighting distinct differences in the transcriptional regulation of virulence factors across different phylogroups. This variability extends to LEE-associated mechanisms, such as pedestal formation mediated by Tir [3].

T3S and differential regulation of virulence factors across strains have also been documented in other pathogens, such as *Pseudomonas aeruginosa*, which uses its T3SS to secrete four virulence effectors (ExoS, ExoT, ExoY and ExoU [71]. Notably, *P. aeruginosa* isolates can show phenotypic differences despite limited genetic variability [72], and the presence or absence of T3S effector genes correlates with clinical outcomes [73,74]. Sublines of the laboratory strain PAO1 exhibit phenotypic variability, particularly in secreted products, despite high genomic similarity, likely due to transcriptional and translational regulation [75]. The secretion of ExoU or ExoS has been linked to reduced cell viability, suggesting that even healthy individuals can carry cytotoxic strains [76]. ExoS has been associated with chronic cystic fibrosis infections, while ExoU is linked to acute invasive infections [77]. Over time, cystic fibrosis isolates show a decline in T3S as infections persist [78], with significant variation in secreted proteins, despite the conservation of intracellular proteins, highlighting secretome expression as a key indicator of strain variation [79].

Further investigation is needed to determine whether strains that secrete poorly or hyper-secrete under the growth conditions assessed in this study exhibit differential LEE gene expression under alternative conditions. The identification of alternative regulatory mechanisms that lead to either constitutive activation or repression of the LEE in strains D3158 and D3309, raises intriguing questions about their adaptive significance in clinical settings. These observations prompt speculation on whether these mechanisms provide advantages within the host environment, while potentially posing drawbacks outside the host due to the energy demands associated with uncontrolled island expression or the restricted ability to engage in host colonization. It is also possible that these strains respond to niche-specific signals within the host, thereby enabling fine-tuned regulation of LEE expression.

This study not only sheds light on alternative mechanisms of LEE regulation but also highlights the ongoing diversification observed in bacterial pathogens. While prototype strains have been pivotal in advancing our understanding of EPEC, they do not fully capture the diversity present in nature nor the adaptability that gives rise to strains potentially more adept in clinical contexts [80].

Although this study provides valuable insights, the number of clinical isolates and growth conditions tested was limited, potentially underrepresenting the full diversity of LEE regulation. Additionally, while the study suggests potential alternative mechanisms of LEE regulation, the molecular pathways remain to be elucidated. Investigating how strain D3158 maintains LEE activation without GrlA and PerC or how strain D3309 succesfully colonizes a host and establishes infection presents exciting avenues for future research. Transcriptional analysis, genomic comparisons, data mining and genetic approaches could be instrumental in identifying novel regulatory elements or pathways driving these processes.

Overall, this work highlights the importance of studying clinical isolates alongside prototype strains to gain a more comprehensive understanding of the diverse virulence strategies employed by EPEC and related pathogens. Expanding these studies to include a broader range of clinical strains could deepen our understanding of the correlation between phenotype and clinical outcomes, further unraveling the complexities of EPEC pathogenesis and its broader impact on patient health.

## supplementary material

10.1099/jmm.0.001907 Uncited Fig. S1.

## Abbreviations

A/E: attaching and effacing
aEPEC: atypical EPEC
BFP: bundle-forming pilus
CAT: chloramphenicol acetyltransferase
DEPC: diethyl pyrocarbonate
DMEM: Dulbecco’s Modified Eagle Medium
EPEC: enteropathogenic *Escherichia coli*
GrlA: global regulator of LEE-activator
GrlR: global regulator of LEE-repressor
LA: localized adherence
LB: lysogeny broth or Luria broth
LEE: locus of enterocyte effacement
Ler: LEE-encoded regulator
PAGE: polyacrylamide gel electrophoresis
pEAF: *E. coli* adherence factor plasmid
PerC: plasmid-encoded regulator
qRT-PCR: quantitative real-time PCR
SDS: sodium dodecyl sulfate
TCA: trichloroacetic acid
tEPEC: typical EPEC
T3S: type III secretion
T3SS: type III secretion system
