# Supplementary figures and images for: Phenotypic diversity of type III secretion system activity in enteropathogenic Escherichia coli clinical isolates
Source: J Med Microbiol. 2024 Oct 21;73(10):001907. doi: 10.1099/jmm.0.001907 (PMC11493143; doi:10.1099/jmm.0.001907)

Figure S1

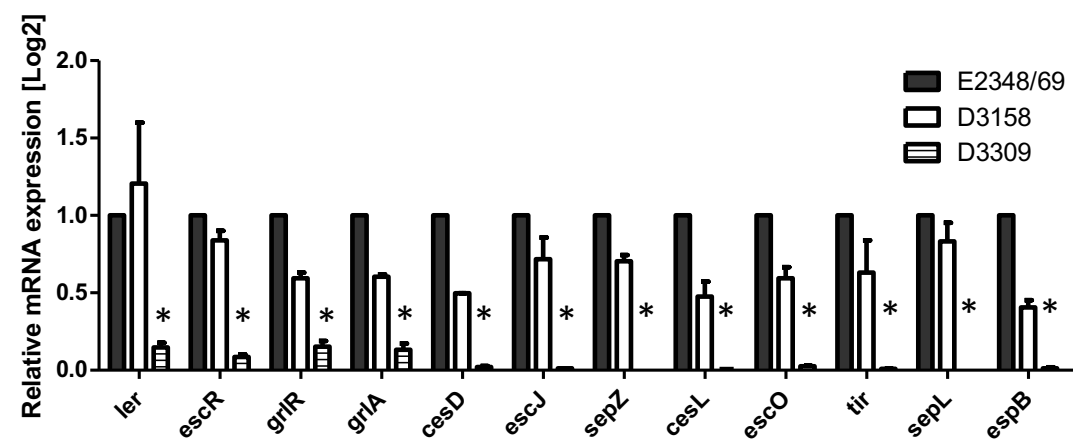

Supplement: Uncited Fig. S1. [file jmm-73-01907-s001.pdf]
